# Contrasting Expression of Canonical Wnt Signaling Reporters *TOPGAL*, *BATGAL* and *Axin2^LacZ^* during Murine Lung Development and Repair

**DOI:** 10.1371/journal.pone.0023139

**Published:** 2011-08-09

**Authors:** Denise Al Alam, Melissa Green, Reza Tabatabai Irani, Sara Parsa, Soula Danopoulos, Frederic G. Sala, Jonathan Branch, Elie El Agha, Caterina Tiozzo, Robert Voswinckel, Edwin C. Jesudason, David Warburton, Saverio Bellusci

**Affiliations:** 1 Developmental Biology and Regenerative Medicine Program, Saban Research Institute of Children's Hospital Los Angeles, Los Angeles, California, United States of America; 2 Excellence Cluster in Cardio-Pulmonary Systems, Department of Internal Medicine II, University of Giessen Lung Center, Giessen, Germany; 3 Lung Development and Remodelling, Max-Planck-Institute for Heart and Lung Research, Bad Nauheim, Germany; 4 Division of Child Health, University of Liverpool, Alder Hey Children's Hospital, Liverpool, United Kingdom; Comprehensive Pneumology Center, Germany

## Abstract

Canonical Wnt signaling plays multiple roles in lung organogenesis and repair by regulating early progenitor cell fates: investigation has been enhanced by canonical Wnt reporter mice, *TOPGAL*, *BATGAL* and *Axin2^LacZ^*. Although widely used, it remains unclear whether these reporters convey the same information about canonical Wnt signaling. We therefore compared beta-galactosidase expression patterns in canonical Wnt signaling of these reporter mice in whole embryo versus isolated prenatal lungs. To determine if expression varied further during repair, we analyzed comparative pulmonary expression of beta-galactosidase after naphthalene injury. Our data show important differences between reporter mice. While *TOPGAL* and *BATGAL* lines demonstrate Wnt signaling well in early lung epithelium, *BATGAL* expression is markedly reduced in late embryonic and adult lungs. By contrast, *Axin2^LacZ^* expression is sustained in embryonic lung mesenchyme as well as epithelium. Three days into repair after naphthalene, *BATGAL* expression is induced in bronchial epithelium as well as *TOPGAL* expression (already strongly expressed without injury). *Axin2^LacZ^* expression is increased in bronchial epithelium of injured lungs. Interestingly, both *TOPGAL* and *Axin2^LacZ^* are up regulated in parabronchial smooth muscle cells during repair. Therefore the optimal choice of Wnt reporter line depends on whether up- or down-regulation of canonical Wnt signal reporting in either lung epithelium or mesenchyme is being compared.

## Introduction

Canonical Wnt signaling plays multiple roles during lung organogenesis and repair by controlling survival, proliferation and differentiation of early progenitor cells in epithelium and mesenchyme [1], [2]. Canonical Wnt signaling is mediated mainly by the multifunctional beta-catenin protein which is a potent co-activator of transcription factors such as Lymphoid Enhancer Factor (LEF) and T Cell Factor (TCF) [3]. Classical s-catenin activation requires binding of secreted lipoglycoproteins termed Wnts to the Frizzled receptor, thereby raising cytoplasmic levels of activated s-catenin, and ultimately nuclear translocation of s-catenin. Without Wnt activation, s-catenin is mainly located at epithelial junctions where it acts as a cell adhesion molecule by interacting with E-cadherin and alpha-catenin [4]. s-catenin has a rapid turnover; excess s-catenin binds to a APC/Axin/GSK3ß complex that mediates its phosphorylation, ubiquitination and degradation [5]. Upon frizzled/LRP5/6 activation, the destruction complex is dismantled by release of *Axin* such that s-catenin is now released, stabilized and translocated into the nucleus (reviewed in [6], [7], [8]).

Several reporter mice have been designed to track Wnt signaling in vivo: two allow monitoring of formation of the s-catenin/TCF transcription complex. The *Tcf* optimal promoter (TOP)-beta-galactosidase (*TOPGAL*) transgenic mice were made fusing three LEF/TCF binding sites to *c-fos* minimal promoter [9]. These mice were originally reported to follow activation of LEF/TCF transcription complexes during hair development and differentiation. The second reporter line, the s-catenin activated transgene (BAT) driving the expression of nuclear beta-galactosidase, was designed by fusing seven TCF/LEF binding sites upstream of a 0.13 kb fragment containing the minimal promoter- TATA box of the *Siamois* gene [10]. However, transgenic mice relying upon random insertion of an expression cassette may be unstable with increased number of generations.

In contrast to the first 2 reporter lines, a stable knock in of *LacZ* in frame with the endogenous start codon of the *Axin2* gene has recently been generated [11]. *Axin2* induces s-catenin degradation [12] in a negative feedback loop controlling Wnt signaling. However *Axin2*, also known as *conductin*, is also a target of the canonical Wnt signaling pathway and its expression can therefore be used to report the activation of this pathway [11].

Herein, we have directly compared reporter expression in the *BATGAL* and *TOPGAL* transgenics, seven and eleven years respectively after their first publication, using the stable *Axin2* for additional comparison. We have tested each reporter not only during development but also during repair after naphthalene induced airway injury. We show the optimal choice of canonical Wnt reporter line depends on whether an up- or down-regulation of canonical Wnt signaling in either lung epithelium or mesenchyme is being evaluated.

## Results

### Embryonic LacZ expression differs significantly between reporter mice

**Figure 1:**
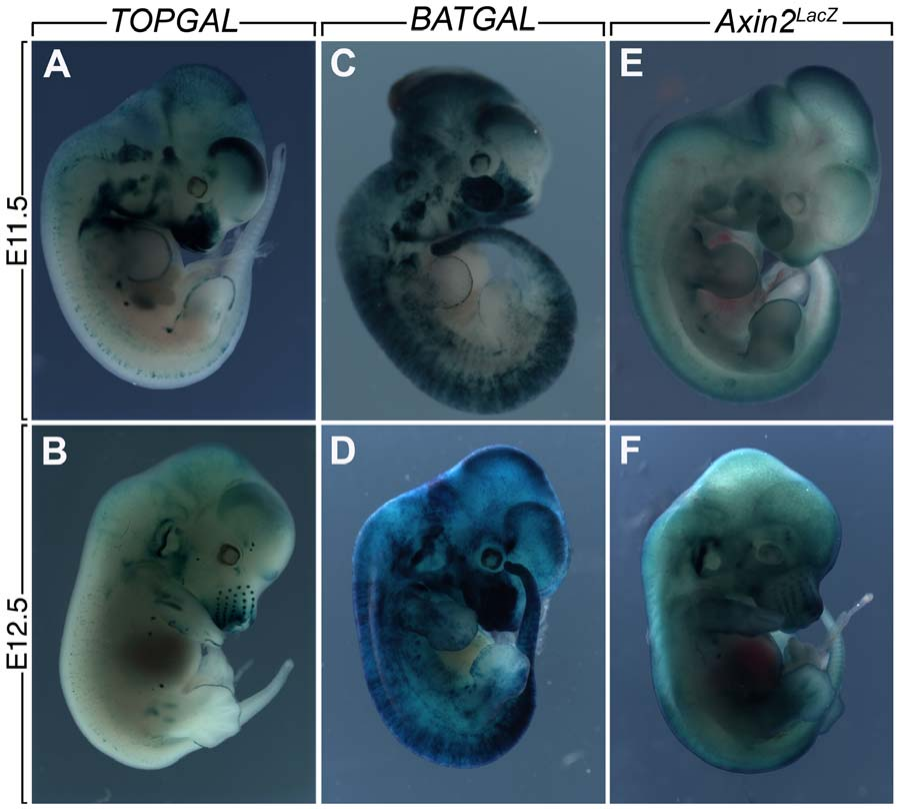
LacZ expression in whole embryos of *TOPGAL*, *BATGAL* and *Axin2^LacZ^* mice. (**A**) E11.5 *TOPGAL* embryo shows staining in the forebrain, the nasal process, the inner ear, the apical ectodermal ridge (AER) of the limb, the epithelium of the mammary placode, the somites and the tip of the tail. (**B**) E12.5 *TOPGAL* embryo shows LacZ expression in ectodermal appendages, the whisker placodes in the nasal region as well as the AER, the mammary buds in between the limbs and discrete mesenchymal condensation within the limbs. (**C**) E11.5 *BATGAL* embryo shows expression throughout the embryo with a “salt and pepper” pattern with higher signals in ectodermal domains such as the nasal process, the forebrain, the AER and the tip of the tail. (**D**) At E12.5, the *BATGAL* embryo still shows “salt and pepper” expression throughout the embryo. LacZ expression is found in the mammary buds but significant staining was also found in the surrounding tissue. (**E**) E11.5 *Axin2^LacZ^* embryo shows homogenous LacZ expression throughout the embryo with higher expression in the AER and the developing mammary placode. (**F**) At E12.5, individualized whisker placodes as well as mammary buds and the AER are clearly positive for *Axin2^LacZ^*. In addition, strong LacZ expression is found in the developing ear and in the somites.

We examined LacZ activity in the whole embryo at E11.5 and E12.5 with a focus on ectoderm-derived organs controlled by Wnt signaling such as whiskers, mammary placodes and limbs. At E11.5 *TOPGAL* is expressed diffusely in the forebrain, nasal process and inner ear. Further specific expression is seen in the apical ectodermal ridge (AER) of the limb, and epithelium of the mammary placode (Fig. 1A). TOPGAL expression is also found in the dorsal and ventral somites as well as the tip of the tail. At E12.5 (Fig. 1B), the expression in ectodermal appendages is maintained and enhanced in the whisker placodes in the nasal region as well as in the AER and the mammary buds. LacZ expression is also detected in discrete mesenchymal condensations within the limbs. In contrast, at E11.5 *BATGAL* expression is found throughout the embryo with a “salt and pepper” pattern (Fig. 1C). LacZ expression is high in ectodermal domains found also to be positive with the TOPGAL line such as the nasal process, the forebrain, the AER and the tip of the tail. At E12.5, “salt and pepper” BATGAL expression throughout the embryo persists. It was not possible to individualize BATGAL expression in the whisker placodes and LacZ expression in the AER was barely detectable (Fig. 1D). LacZ expression was found in the mammary buds but significant staining was also found in the surrounding tissue. We also examined expression of *Axin2^LacZ^* (Fig. 1E–F). At E11.5, in contrast to the salt and pepper expression of *BATGAL*, *Axin2* is expressed homogeneously throughout the embryo except in the AER and the developing mammary placode, where higher levels of expression are found. At E12.5, individualized whisker placodes as well as mammary buds and the AER are clearly positive for *Axin2*. In addition, strong LacZ expression is found in the developing ear as well as in the somites. LacZ expression is also found in the developing vascular network underneath the skin. We next examined the expression of LacZ in the developing lung.

### Sustained *TOPGAL* and *Axin2^LacZ^* expression contrasts with decreased *BATGAL* expression in developing lung

**Figure 2:**
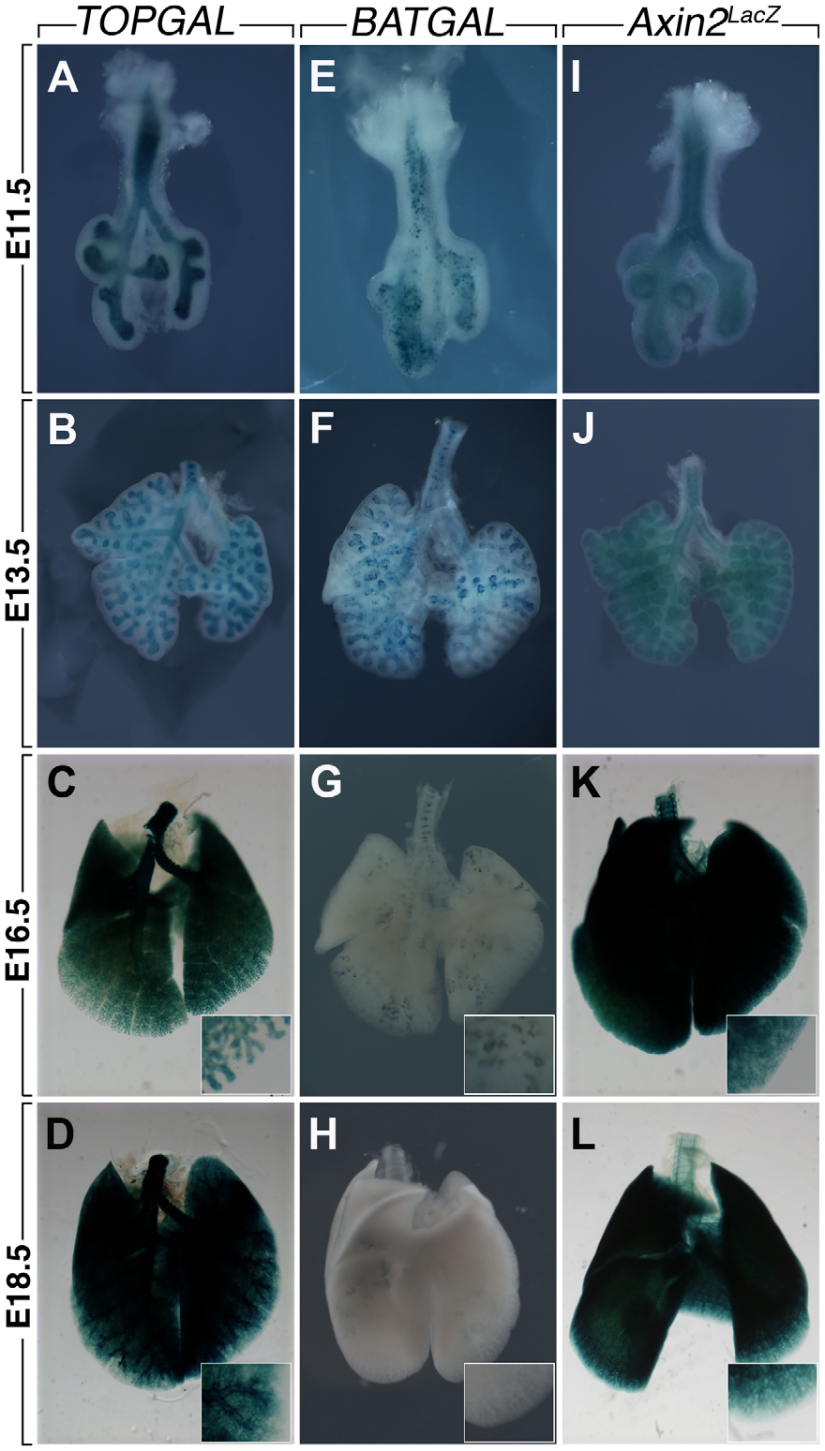
LacZ expression in *TOPGAL*, *BATGAL* and *Axin2^LacZ^* whole lungs during prenatal development. *TOPGAL* is expressed in the epithelium of the trachea and the lung at E11.5 (**A**), E13.5 (**B**) and E16.5 (**C**); whereas at E18.5 (**D**) *TOPGAL* expression is also detected in the terminal bronchioles and the surrounding parenchyma. *BATGAL* expression is detected in a “salt and pepper” manner in the lung epithelium at E11.5 (**E**) and E13.5 (**F**). This expression is drastically reduced at E16.5 (**G**) and E18.5 (**H**). *Axin2^LacZ^* expression is found in both the epithelium and mesenchyme at E11.5 (**I**), E13.5 (**J**), E16.5 (**K**) and e18.5 (**L**).

**Figure 3:**
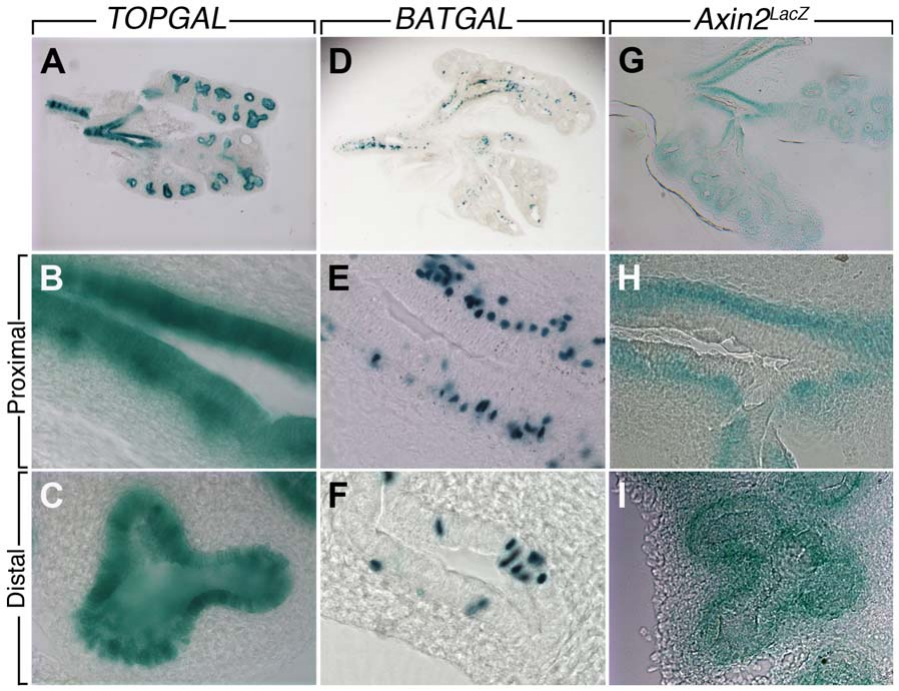
Vibratome sections of E13.5 *TOPGAL*, *BATGAL* and *Axin2^LacZ^* lungs. *TOPGAL* is expressed in both epithelium and mesenchyme at the level of the bronchi (**A, B**) and restricted to the epithelium in the distal lung (**C**). *BATGAL* “salt and pepper” expression is found in a heterogeneous fashion in the lung (**D**). The expression is restricted to the mesenchyme adjacent to the bronchial epithelium (**E**). In the distal lung, *BATGAL* is sporadically expressed in the epithelium (**F**). *Axin2^LacZ^* is found exclusively in the mesenchyme adjacent to the epithelium at the bronchial level (**G, H**). While in the distal lung, LacZ expression is found mainly in the epithelium and at lower level in the mesenchyme (**I**).

Mouse embryonic lungs at early, mid and late pseudoglandular stages (E11.5, E13.5 and E16.5) as well as at the saccular stage (E18.5) were isolated and examined for LacZ expression. At E11.5, E13.5 and E16.5 TOPGAL expression is mostly detected in the epithelium of the proximal and distal lung as well as in the trachea (Fig. 2A–C). At E18.5, LacZ expression is detected in the terminal bronchioles as well as the surrounding parenchyma (Fig. 2D). In contrast, at E11.5, BATGAL expression is detected in a “salt and pepper” fashion in the lung epithelium (Fig. 2E). This expression is drastically reduced at the other stages examined. Only patches of LacZ expression are observed (Fig. 2F–G). At E18.5, there is almost no LacZ expression detectable (Fig. 2H). We finally examined the expression of *Axin2^LacZ^* (Fig. 2I–L). At E11.5, LacZ expression is found in the lung epithelium with lower levels in the mesenchyme. In the trachea and the primary bronchi, it appears that the expression is in both the epithelium and the adjacent mesenchyme. At E13.5, this expression pattern is maintained with the exception of the primary bronchi where LacZ is now expressed only in the mesenchyme as a ring-like structure. At E16.5, LacZ expression is found in both the epithelium and mesenchyme. Such expression is maintained at E18.5. Vibratome sections of the E13.5 lungs were carried out to better visualize the presence of LacZ expression in the bronchi and the distal lung. In the bronchi, LacZ expression due to *TOPGAL* is found in the epithelium and mesenchyme (Fig. 3A,B), however, in the distal lung, LacZ expression is only found in the epithelium (Fig. 3C). *BATGAL* “salt and pepper” expression is found in a heterogeneous fashion in the lung (Fig. 3D). In the mesenchyme, *BATGAL* expression is restricted to the mesenchyme adjacent to the bronchial epithelium (Fig. 3E) and is sporadically present in the distal lung epithelium (Fig. 3F). Similar to *BATGAL* expression in the bronchi, *Axin2^LacZ^* is found exclusively in the mesenchyme adjacent to the epithelium (Fig. 3G,H). In the distal lung, *Axin2^LacZ^* expression is found mainly in the epithelium but it is also present at lower levels in the mesenchyme (Fig. 3I).

### *Axin2^LacZ^* is a superior line to assess the response to naphthalene injury

**Figure 4:**
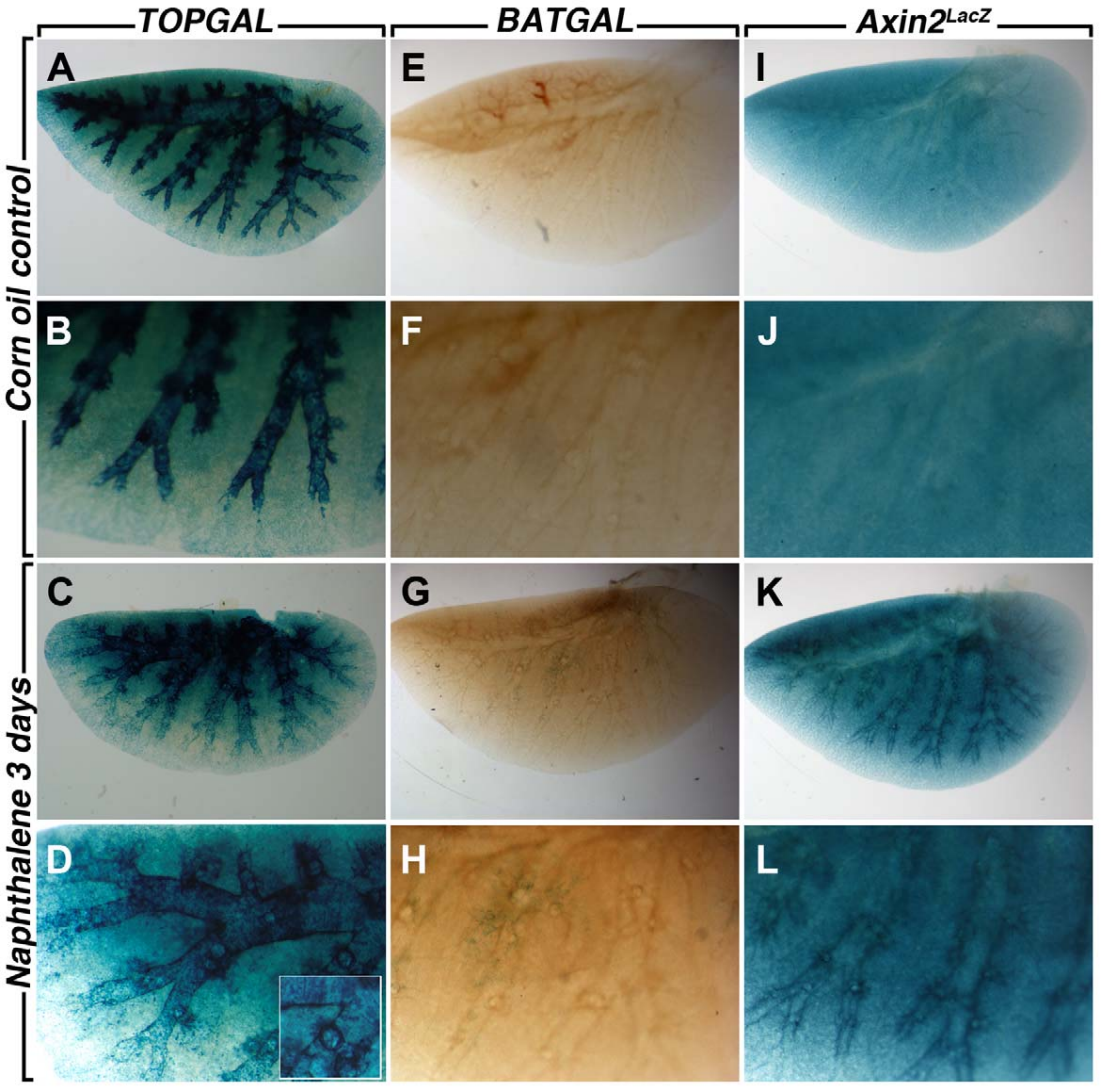
Corn oil and Naphthalene-treated whole mount left lungs cleared with Benzyl benzoate. In the adult lung, *TOPGAL* is strongly expressed in the epithelium (**A, B**) and decreases slightly after naphthalene injury (**C, D**). *BATGAL* expression is totally absent in the adult lung (**E, F**) and a discrete expression appears after injury (**G, H**). While a homogenous *Axin2^LacZ^* expression is present throughout the adult lung (**I, J**), a marked increase surrounding the bronchial epithelium is observed after injury (**K, L**).

**Figure 5:**
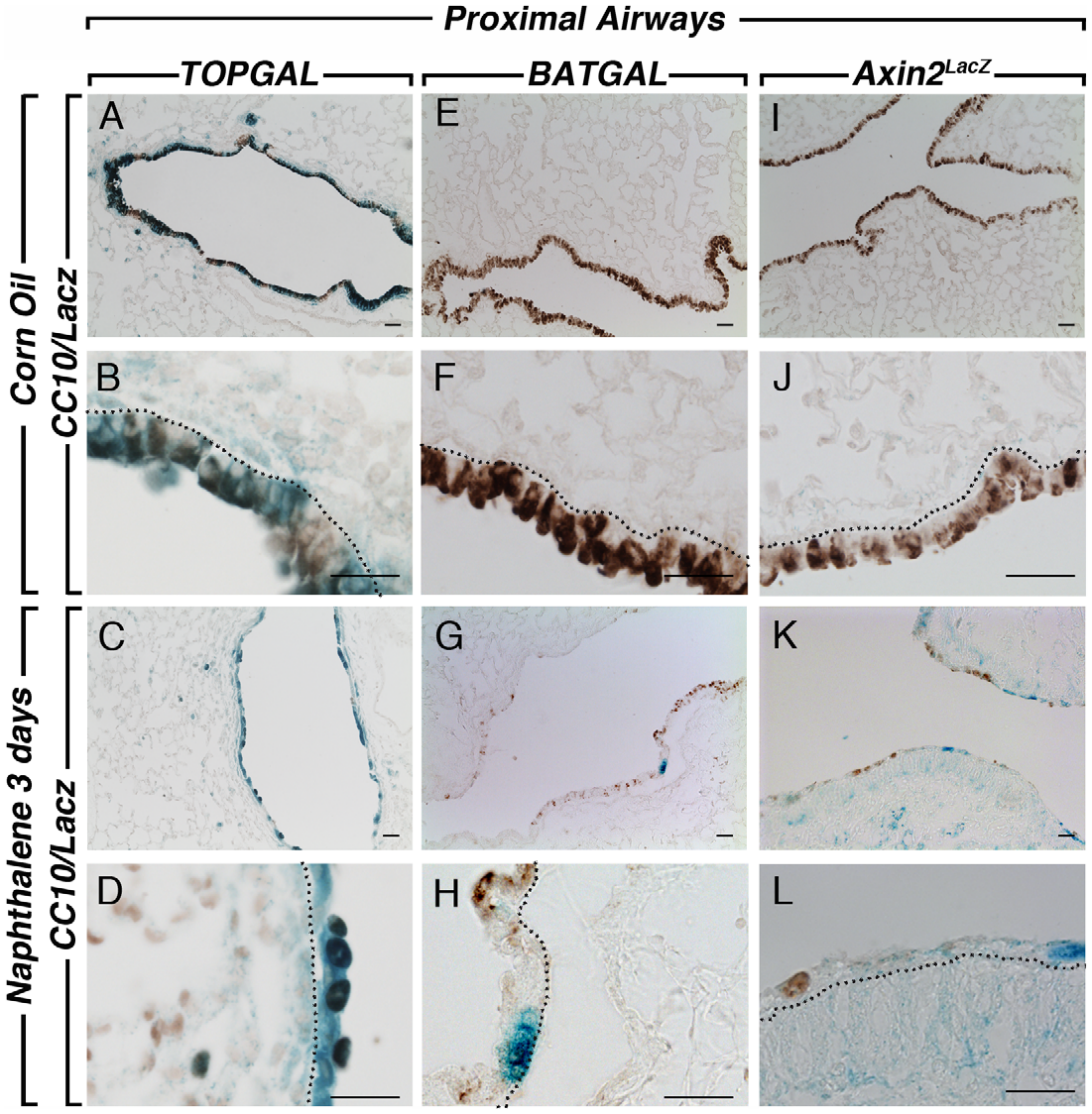
CC10 and LacZ staining in the proximal lungs after naphthalene injury. CC10 and LacZ co-staining in *TOPGAL* control lungs at low (**A**) and high (**B**) magnification show strong expression of *TOPGAL* in the CC10-positive cells. A decrease in the Clara cells after naphthalene injury is shown in the proximal airways (**C, D**). *BATGAL* expression is not detected in the airways (**E, F**) but at discrete spots in the bronchial epithelium after injury (**G, H**). *Axin2^LacZ^* sections showed very low level staining throughout the lung in the conducting airways of the control adult lungs (**I, J**), and an increased expression after injury (**K, L**). Scale bars are 100 µm.

**Figure 6:**
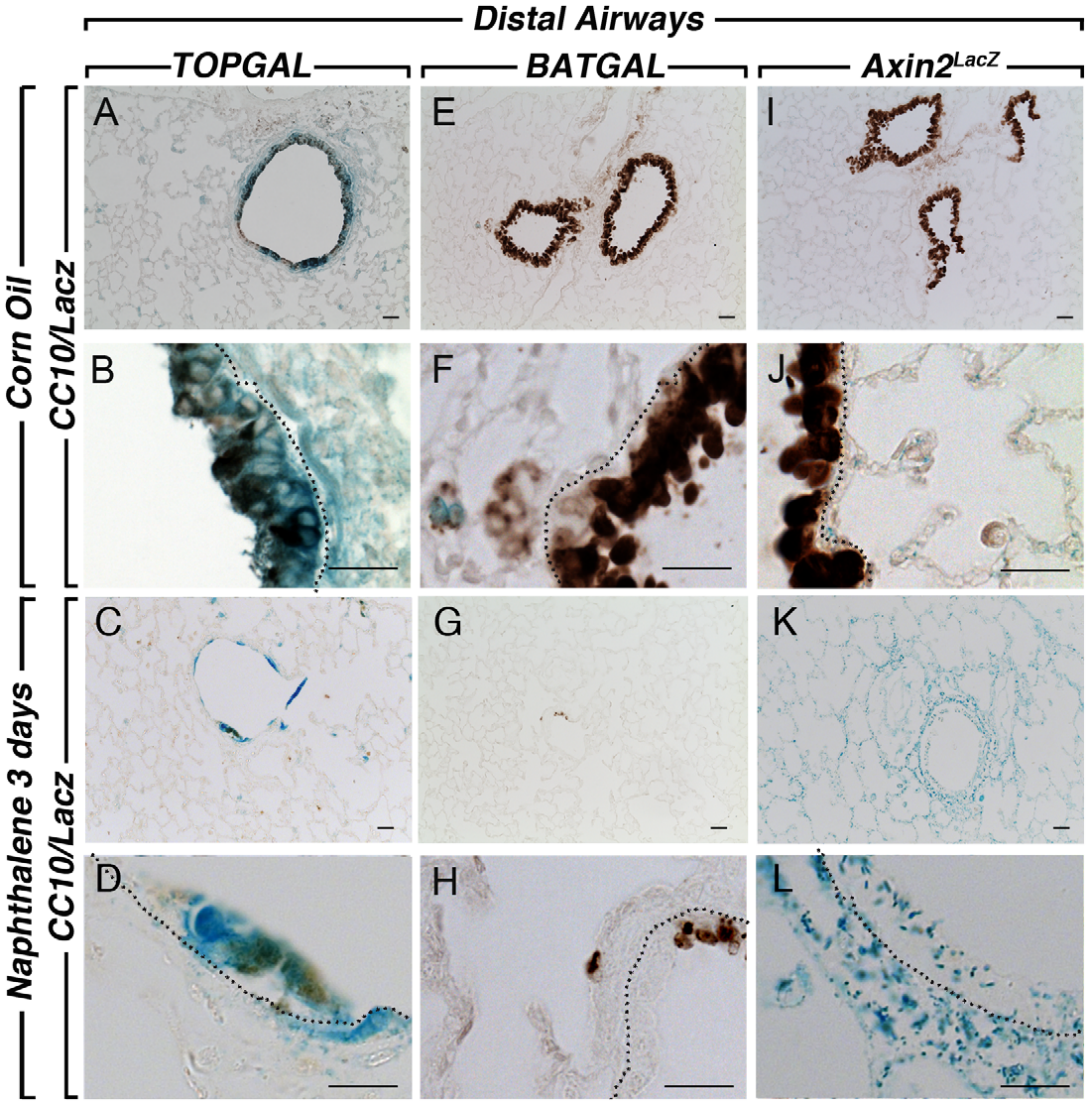
CC10 and LacZ staining in the distal lung compartment after naphthalene injury. CC10 and LacZ co-staining in *TOPGAL* control lungs at low (**A**) and high (**B**) magnification show strong expression of *TOPGAL* in the CC10-positive cells. A decrease in the Clara cells after naphthalene injury is shown in the distal airways (**C, D**). *BATGAL* expression is not detected in the distal airways (**E, F**) of adult control lungs, and still absent from the epithelium (**G, H**) in the distal compartment of the naphthalene-treated lungs. *Axin2^LacZ^* sections showed very low level staining in the distal airways of the control adult lungs (**I, J**), and an increased expression after injury (**K, L**). Scale bars are 100 µm.

**Figure 7:**
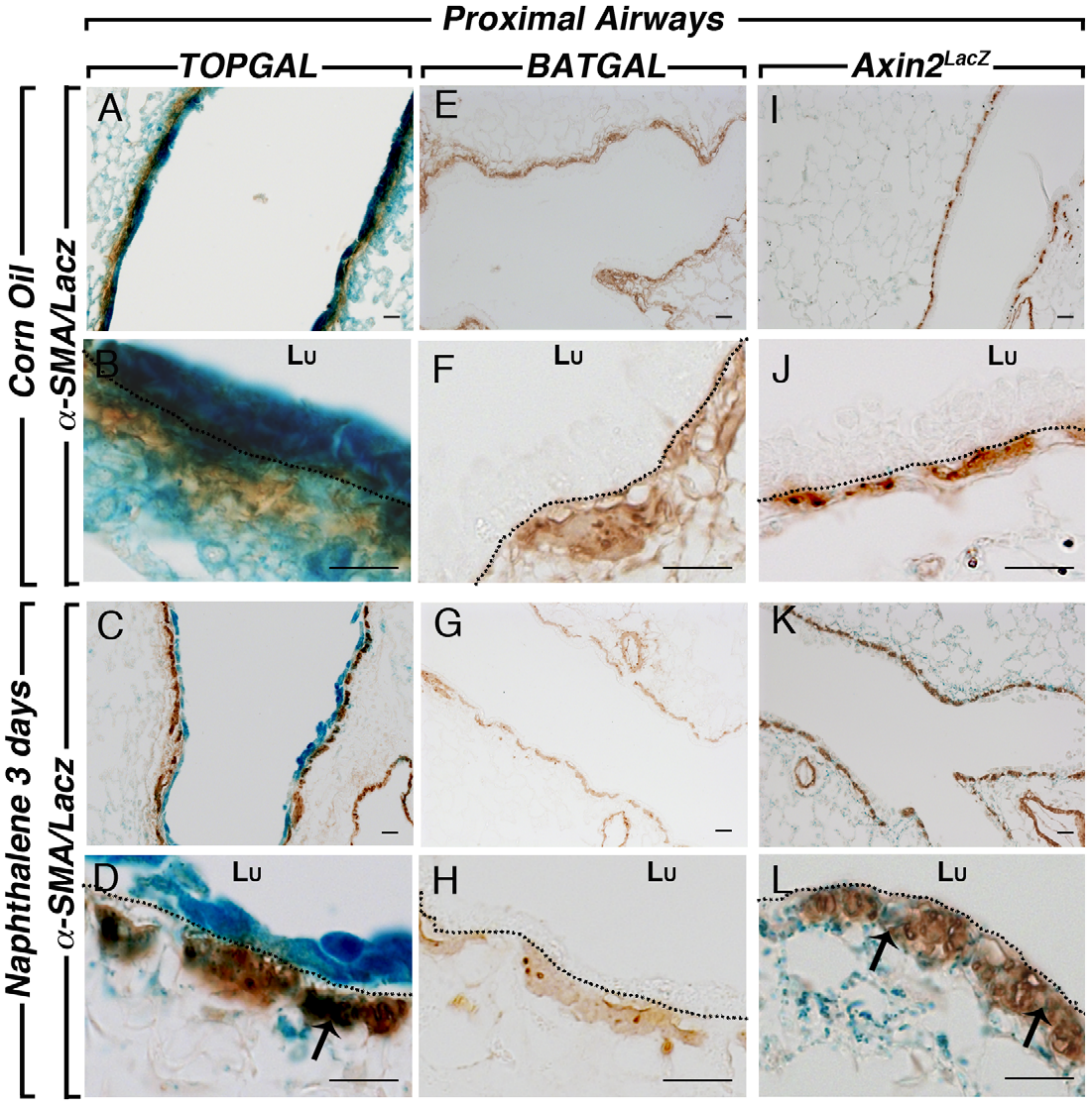
SMA and LacZ staining in the proximal lung compartment after naphthalene injury. Co-staining of SMA and LacZ at low (**A**) and high (**B**) magnifications showed co-localization in the PBSMCs of control and naphthalene-treated lungs (**C, D**). *BATGAL* expression is not detected in the proximal PBSMCs in the control lung (**E, F**) and naphthalene-treated lungs (**G, H**). *Axin2^LacZ^* sections showed low level staining in the PBMSCs of the control adult lungs (**I, J**) whereas LacZ expression is drastically upregulated after injury as pointed out with the arrows (**K, L**). Dotted lines show the basal membrane separating the epithelium from the PSMC layer. Scale bars are 100 µm.

**Figure 8:**
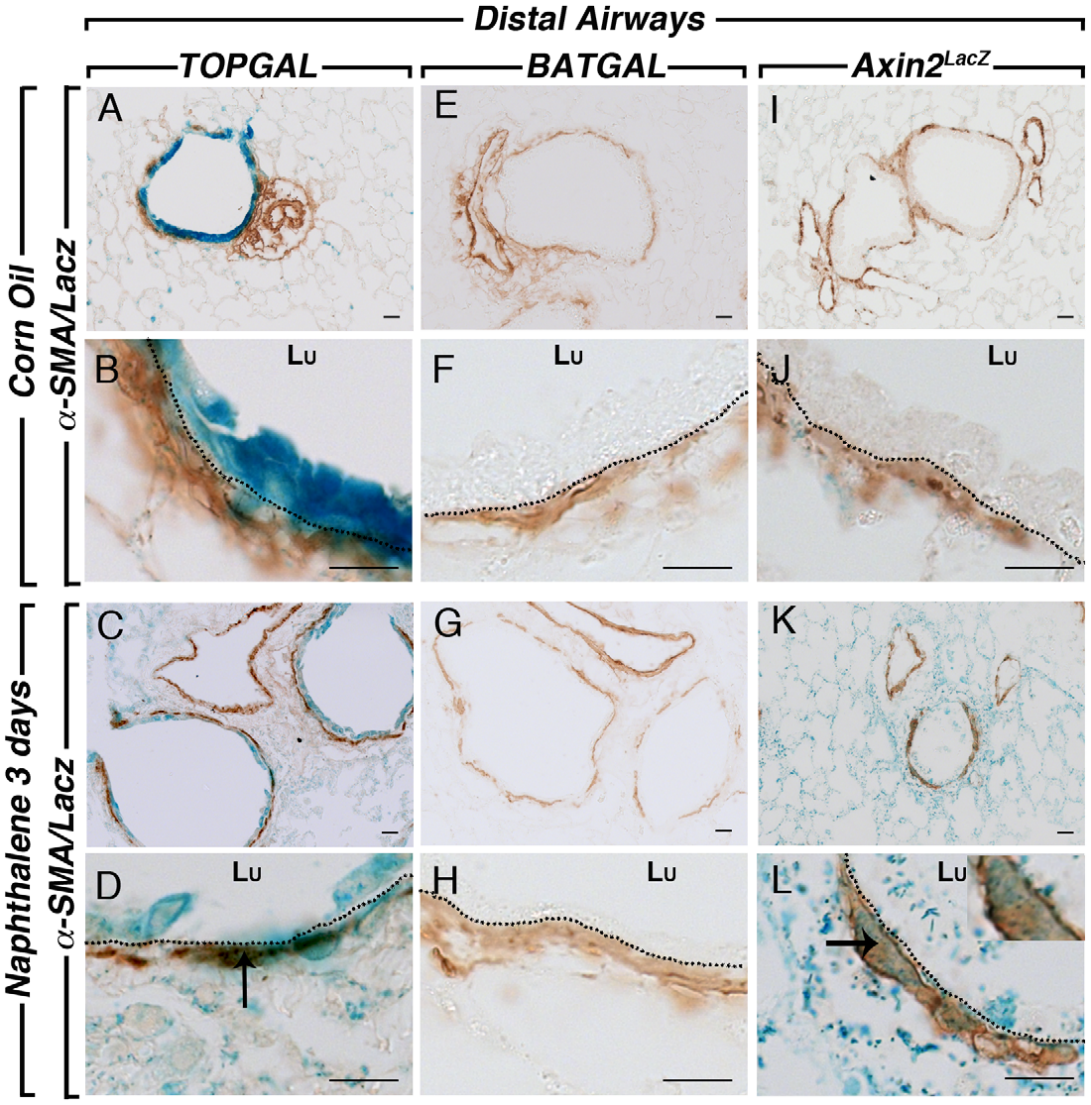
SMA and LacZ staining in the distal lung compartment after naphthalene injury. SMA and LacZ co-staining in *TOPGAL* control lungs at low (**A**) and high (**B**) magnification showed co-localization in the PBSMCs of control (**A, B**) and naphthalene-treated lungs (**C, D**). *BATGAL* expression is not detected in the PBSMCs of adult control lungs (**E, F**) and naphthalene-treated lungs (**G, H**). *Axin2^LacZ^* sections showed low level staining in the PBSMCs surrounding the distal bronchioles in control adult lungs (**I, J**) and an increase after injury (**K, L**). The arrows show co-localisation of SMA and LacZ. Dotted lines show the basal membrane separating the epithelium from the PSMC layer. Scale bars are 100 µm.

We next examined the response of the bronchial airway epithelium of the different Wnt reporter lines to naphthalene, a simple and robust model of lung injury. Corn oil was used as control. Only females were used for the naphthalene injury since females are more susceptible to this type of injury than males [13]. In the conducting airway, naphthalene caused significant epithelial cell death within 12 hours followed by re-epithelization of the airways, presumably by the P450^neg^ variant of Clara cells [14], [15]. Three days after injury onset, the epithelium is in part re-populated, with complete re-epithelialisation 10–14 days after injury [14], [16]. We first visualized the impact of the injury on whole left lung of the Wnt reporter mice studied herein. *TOPGAL* expression in corn oil control shows a high baseline of LacZ expression in the bronchial epithelium (Fig. 4A, B). Since the epithelium was injured and sloughed off, this expression in the epithelium is significantly decreased in the naphthalene-treated lungs (Fig. 4C, D). LacZ expression is now detected in a stripe-like pattern around the bronchi in the parabronchial smooth muscle cells surrounding the damaged conducting airways (inset in Fig. 4D), suggesting a possible role of these cells in the repair process. In contrast, *BATGAL* expression is not detected in the control lung (Fig. 4E, F) and a slight but significant increase in LacZ expression is observed in the bronchial epithelium of naphthalene-treated lung (Fig. 4G, H). *Axin2^LacZ^* expression is detected homogeneously throughout the corn oil control lung in both the conducting and respiratory airways (Fig. 4I,J). In the naphthalene-treated lung, LacZ expression is sharply upregulated in both areas of the lung (Fig. 4K,L). To confirm that the naphthalene injury occurred in our samples, we carried out CC10 staining to follow the Clara cells in control and experimental lungs. Typical robust CC10 expression was present in the corn oil treated lungs of *TOPGAL* (Fig. 5A,B and Fig. 6A,B), *BATGAL* (Fig. 5E,F and Fig. 6E,F) and *Axin2^LacZ^* (Fig. 5I,J and Fig. 6I,J) in both proximal (Fig. 5) and distal airways (Fig. 6). Whereas, Clara cells in naphthalene-treated lungs were severely damaged in the three reporter lines as indicated by the localized expression of CC10 in the proximal (Fig. 5C,D; G,H; K,L) and distal airways (Fig. 6C,D, G,H, K,L). Consistent with the whole mount data, *TOPGAL* was expressed in proximal (Fig. 5A,B) and distal epithelium (Fig. 6A–B) in control lung on LacZ/CC10 staining, as well as in the parabronchial smooth muscle cells (PBSMCs) at both proximal (Fig. 7A,B) and distal (Fig. 8A,B) levels as indicated by SMA staining. While CC10 staining decreased drastically in the naphthalene-treated *TOPGAL* lungs, *TOPGAL* expression is present in the CC10-positive cells re-populating the airways mainly in the proximal compartment (Fig. 5C,D) as compared to the distal compartment (Fig. 6C,D). Moreover, *TOPGAL* expression was still present in the PBSMCs surrounding the proximal (Fig. 7C,D) and distal airways (Fig. 8C,D). Moreover, *BATGAL* expression is not detected in control lungs at the proximal (Fig. 5E,F) or distal level (Fig. 6E,F). However, *BATGAL* is found at discrete spots in the bronchial epithelium of naphthalene-treated lungs (Fig. 5G,H) and is still completely absent in the distal airways (Fig. 6G,H) and in the PBSMCs (Fig. 7G,H and Fig. 8G,H). Finally, *Axin2^LacZ^* expression is found at low level throughout the lung in both the epithelium (Fig. 5I,J and Fig. 6I,J) and the PBSMCs (Fig. 7I,J and 8I,J) in proximal and distal airways. An increase of LacZ expression is observed in both proximal (Fig. 5K,L) and distal epithelium (Fig. 6K,L) of naphthalene-treated lungs as well as in the PBSMCs surrounding proximal (Fig. 7K,L) and distal airways (Fig. 8K,L). The arrows displayed in Fig. 7 and 8 show co-localization of SMA and LacZ after naphthalene injury in *TOPGAL* and *Axin2^LacZ^* mice. qRT-PCR analyses showed a 10-fold increase in beta-galactosidase expression in the *Axin2^LacZ^* naphthalene-treated lungs as compared to controls accompanied with a 8-fold decrease in *CC10* expression (data not shown).

## Discussion

Our aim was to provide a systematic comparison of the expression patterns of three different but classical Wnt reporter lines that are in common use and on whose output depend a growing number of published research findings. We exploited the stable *Axin2^LacZ^* line to provide an internal control for the older *TOPGAL* and *BATGAL* lines where random insertion may alter expression from the original reports (both first made >6 years ago). We chose to use the developing lung and the repairing lung as model systems in which to test such expression since both scenarios require canonical Wnt signaling. Our findings can be a significant resource to not only the lung field but also the many other bioscience research areas in which Wnt function is being investigated. Crosses to introduce one of these reporter alleles in already complex combinations of driver and responder lines is resource and time intensive but our findings can guide the critical selection of the appropriate Wnt reporter line.

Our data indicate that *Axin2^LacZ^* mice are the Wnt reporter line of choice for the specific detection of increased Wnt signaling in the epithelium. Surprisingly, only one paper so far makes use of this line to follow Wnt signaling in vivo in the lung [17]. Most of the papers published to date make extensive use of *TOPGAL* mice. The usefulness of the *BATGAL* mice to detect an increase in Wnt signaling is due to the progressive disappearance of LacZ signal as the embryo develops with almost complete absence of LacZ expression in the E18.5 lungs. Similar results are observed in the lungs of 2 month-old *BATGAL* mice. In harmony with these results, these mice have been used to demonstrate that a Gata6-Wnt pathway is required for epithelial stem cell development and airway regeneration [18]. Gata6 is a transcription factor, which negatively regulates the canonical Wnt pathway. Inactivation of *Gata6* in the lung epithelium in the background of the *BATGAL* allele leads to a drastic increase in LacZ expression in the epithelium at E13.5 and E16.5 demonstrating the negative role played by Gata6 on the activation of Wnt signaling. The same authors also reported the progressive appearance of *BATGAL* positive cells, starting at 2 days post-naphthalene injury, in the broncho-alveolar duct junction of the airways. Our results concerning the use of *BATGAL* are therefore in agreement with this report.

The *TOPGAL* reporter has been used most extensively, probably because it was the first published line made available to monitor Wnt signaling in vivo. During development, *TOPGAL* expression was present in epithelium of the developing lung from E11.5 to E18.5, with higher expression in proximal compared to distal epithelium [19]. This line has mainly been used to demonstrate reduced canonical Wnt signaling. For example this line was used to validate the biological activity of recombinant Dickkopf-1, a canonical Wnt inhibitor [20], to show that deletion of R-Spondin leads to reduction in *TOPGAL* reporter activity [21] and to demonstrate reduction of Wnt signaling in elastase and cigarette smoke-induced lung emphysema [22]. Moreover, an increase in *TOPGAL* expression was reported in hyperoxia injury model in the lungs of neonate mice [23].

Our results with *Axin2^LacZ^* mice indicate that this line is the most robust and faithful line to detect Wnt signaling. As expected for such a critical pathway, this line indicates that Wnt signaling occurs throughout lung development as well as in the adult lung, in both the epithelium and the mesenchyme, in both the conducting and respiratory airways. Harris-Johnson et al. showed that *Axin2^LacZ^* is expressed early on (E9.5) in the prospective respiratory region and it is restricted to the ventral foregut that will form the lung and trachea [24]. This line has also been used by another group reporting that adult alveolar type II cells do not exhibit constitutive beta-catenin signaling in vivo. However, after bleomycin injury, a significant increase in the number of LacZ/SPC double positive cells is observed [17]. We also showed increased expression of *Axin2^LacZ^* in response to naphthalene injury in adult mice, and in response to hyperoxia injury in neonates (data not shown). Although, this line will be used more extensively in the future, an important inherent limitation of the Axin2^LacZ^ line is the corresponding up-regulation of endogenous Wnt signaling, since the Axin-related protein, AXIN2, modulates beta-catenin stability. Deregulation of beta-catenin is important in the genesis of several malignancies and this aspect will therefore have to be taken into consideration. The *Axin2^lacZ^* mice therefore represent more than just stable reporters for Wnt signaling; they are also a gain of function of canonical beta-catenin signaling, both in the epithelium and mesenchyme. This line has been used with success to demonstrate that Wnt signaling allows self-renewal of mammary stem cells and promotes their long-term propagation in culture [25].

In conclusion, our data indicate that the choice of the appropriate Wnt reporter line should be tailored to the need to detect either up- or down-regulation of the canonical Wnt signaling pathway in the lung epithelium versus the mesenchyme. Until better tools to follow Wnt signaling are available (e.g. a transgenic construct with *Axin2* regulatory sequences upstream of LacZ), this conclusion is highly relevant to the wide range of research fields studying Wnt signaling. Moreover, our findings represent a reference resource for researchers pursuing such work within the pulmonary field and beyond.

## Materials and Methods

### Mice

The 3 reporter mice used in this study were obtained from the Jackson lab. *TOPGAL* mice (Tg(Fos-lacZ)34Efu/J, stock number 004623) were generated by Das Gupta and Fuchs in 1999. *BATGAL* mice (B6.Cg-Tg(BAT-lacZ)3Picc/J, 005317) were generated by Maretto et al. in 2003. *Axin2^LacZ^* (B6.129P2-*Axin2^tm1Wbm^*/J, stock number 009120) were generated by Lustig et al. in 2002 [11]. BATGAL and *Axin2^LacZ^* lines purchased from the Jackson lab are on a C57 black (C57BL) background while *TOPGAL* mice are on CD1 background. To eliminate any background variabilities, we backcrossed the *TOPGAL* line with C57BL for more than 6 generations to obtain a pure C57BL background. Animal experiments were performed under the research protocol approved by the Animal Research Committee at Children's Hospital Los Angeles and in strict accordance with the recommendations in the Guide for the Care and Use of Laboratory Animals of the National Institutes of Health. The approval identification for Children's Hospital Los Angeles is AAALAC A3276-01. These experiments were done under the protocol 31-08.

### X-gal staining

Mouse embryos were isolated at E11.5 and E12.5 in Hank's solution, washed briefly in PBS, pre-fixed for 10 min with 4% PFA and washed twice in LacZ buffer solution. Embryos were incubated overnight at 37°C with the LacZ buffer solution containing 40 mg/mL of X-gal (rpi research products). Embryonic lungs were dissected out in DMEM from embryos at E11.5, E13.5, E16.5 and E18.5. Lungs were washed briefly in PBS and pre-fixed for 10 min in 4% PFA before incubation overnight with the X-gal solution. Vibratome lung sections 20 µm thickness were carried out at E13.5. Three independent litters for each time point were collected. Littermates that do not carry the beta-galactosidase were used as controls and did not show any LacZ staining.

For adult lungs, transcardiac perfusion with PBS was performed to remove the red blood cells in the lung. The lungs were inflated trans-tracheally at 25 cm of water pressure and submerged in 4% PFA for 5 min, washed 5 minutes in PBS and 5 minutes in LacZ buffer. The lungs were then inflated again with the LacZ buffer containing 40 mg/mL of X-gal. The trachea was ligated using sutures to maintain the staining solution inside the lung. The whole lung was then incubated in 10 mL of staining solution overnight at 37°C. The lungs were subsequently washed with PBS and fixed again in 4% PFA in PBS at room temperature overnight. For better visualization of the staining inside the lung, lungs were dehydrated and cleared with BABB (1∶2 Benzyl Alcohol and Benzyl Benzoate). Dehydrated lungs were transferred to 1∶2 BABB and ethanol for 20 min, 2∶1 BABB and ethanol for 20 min, and 100% BABB for 20 min.

### Naphthalene injury

Naphthalene, NA (Fisher, Aschaffenburg, Germany) was dissolved in corn oil at 30 mg/mL. *TOPGAL*, *BATGAL* and *Axin2^LacZ^* 2 month-old ice were injected IP with 250 mg/kg body weight of either naphthalene or the same volume of a vehicular control of corn oil alone and the mice were sacrificed 3 days later. For *TOPGAL* and *BATGAL*, 4 adult females were injected with corn oil and 4 adult females were injected with naphthalene. For *Axin2^LacZ^*, 8 adult females were used for control group and 8 for experimental group. The time-point of 3 days was chosen because experiments studying the kinetics of naphthalene-induced acute airway injury revealed that epithelial cells in the conducting airways are proliferating to repair the damaged bronchial epithelium between 2–4 days. It has been reported that complete exfoliation was observed 24 h after naphthalene injection.

### Immunohistochemistry

For microtome sections, after 4% PFA fixation, lungs were washed in PBS, dehydrated, and embedded in paraffin. Sections were performed at 5 microns. The sections were cleared with 2 changes of xylene and hydrated in successive graded Ethanol solutions, equilibrated in water then washed in 3% H_2_O_2_ for 20 min at room temperature. The sections were incubated with primary antibodies anti-CC10 (santa Cruz, 1∶200 dilution) and anti-alpha smooth muscle actin (Dako cytomation, 1∶200 dilution) at 4°C overnight. Immunohistochemistry was performed using Dako EnVision Kit following the manufacturer's instructions. Slides were mounted using xylene-based mounting media. Brightfield images were acquired on an Axio Observer.Z1 microscope equipped with an AxioCam MRc color CCD camera (Carl Zeiss Microimaging, Thornwood, NY). Microscope control and image processing were done with AxioVision 4.8.1.0 software (Carl Zeiss). Images at different magnifications were acquired with the following objective lenses: 20x/0.8 Plan-APOCHROMAT, 40x/1.3 Plan-NEOFLUAR oil immersion, and 63x/1.4 Plan-APOCHROMAT oil immersion (Carl Zeiss). For some fields of view several focus planes were acquired and an Extended Focus projection is shown. Where larger areas were needed four adjacent fields of view were acquired and stitched together with the MosaicX module of the software. CC10 staining was performed on each adult animal included in this study to verify that the injury did occur.

### Real time PCR

RNA was extracted from *Axin2^LacZ^* lungs treated with naphthalene or corn oil for 3 days. RNA was reverse-transcribed into cDNA using Transcriptor High Fidelity cDNA Synthesis Kit (Roche Applied Science) according to the manufacturer's instructions. cDNA was used for dual color Hydrolysis Probe – Universal probe library based real time PCR, using the LightCycler 480 from Roche Applied Science. GAPDH assay commercially available from Roche Applied Science was used as reference gene. The primers and probes used for CC10 and beta-galactosidase are as follows: CC10 Left 5′-gatcgccatcacaatcactg-3′; Right 5′-cagatgtccgaagaagctga-3′ with probe #56 (Roche Applied Science UPL); beta-galactosidase: Left 5′-atggatgagcagacgatgg-3′; Right 5′-cggcgttaaagttgttctgc-3′ with probe #18 (Roche Applied Science).
